# Applying a Complex Integrated Method for Mapping and Assessment of the Degraded Ecosystem Hotspots from Romania

**DOI:** 10.3390/ijerph182111416

**Published:** 2021-10-29

**Authors:** Sorin Avram, Irina Ontel, Carmen Gheorghe, Steliana Rodino, Sanda Roșca

**Affiliations:** 1 National Institute for Economic Research “Costin C. Kiriţescu” (INCE), Romanian Academy, 13 September Street No. 13, 050711 Bucharest, Romania; avram.sorin@ucv.ro (S.A.); carmen.adriana@ince.ro (C.G.); 2 Department of Geography, University of Craiova, Al. I. Cuza Street No. 13, 200585 Craiova, Romania; 3 Remote Sensing and Satellite Meteorology Laboratory, National Meteorological Administration, 013686 Bucharest, Romania; irina.ontel@meteoromania.ro; 4 National Institute of Research and Development for Biological Sciences, Spl. Independentei No. 296, 060031 Bucharest, Romania; steliana.rodino@yahoo.com; 5 Institute of Research for Agriculture Economy and Rural Development, Bd. Marasti No. 61, 011464 Bucharest, Romania; 6 Faculty of Geography, Babes-Bolyai University, 400006 Cluj-Napoca, Romania

**Keywords:** degraded ecosystems, terrestrial ecosystems, freshwater ecosystems, marine ecosystems, Romania

## Abstract

To meet the global challenges of climate change and human activity pressure on biodiversity conservation, it has become vital to map such pressure hotspots. Large areas, such as nation-wide regions, are difficult to map from the point of view of the resources needed for such mapping (human resources, hard and soft resources). European biodiversity policies have focused on restoring degraded ecosystems by at least 10% by 2020, and new policies aim to restore up to 30% of degraded ecosystems by 2030. In this study, methods developed and applied for the assessment of the degradation state of the ecosystems in a semi-automatic manner for the entire Romanian territory (238,391 km^2^) are presented. The following ecosystems were analyzed: forestry, grassland, rivers, lakes, caves and coastal areas. The information and data covering all the ecoregions of the Romania (~110,000 km^2^) were analyzed and processed, based on GIS and remote sensing techniques. The largest degraded areas were identified within the coastal area (49.80%), grassland ecosystems (38.59%) and the cave ecosystems (2.66%), while 27.64% of rivers ecosystems were degraded, followed by 8.52% of forest ecosystems, and 14.05% of lakes ecosystems. This analysis can contribute to better definition of the locations of the most affected areas, which will yield a useful spatial representation for future ecological reconstruction strategy.

## 1. Introduction

The evaluation of the state of ecosystems, as the fundamental structural and functional unit of living matter, is a constant concern of global and European policies, in order to establish guidelines for preventing the loss of their functions. The assessment of the condition of ecosystems necessitates extensive analysis of their physical, chemical and biological quality at a particular moment and measurement of the impacts of major pressures that are arising. Natural ecosystems are constantly exposed to pressures from over-exploitation of resources, extensive hunting, climate change and pollution [1,2]. Some authors consider that the highest direct impact on an ecosystem’s state is represented by anthropogenic pressures (overharvesting and land use change) leading to biodiversity loss [3].

To accurately evaluate the ecosystem services provided by a particular area, first, the state of the ecosystem must be studied. The state of the ecosystem is the first level in the flow of services from nature to society [4], and it defines the ability of the ecosystem to provide services. Pressures from human activity, such as pollution or overuse, can have an impact on the state of the ecosystem, thus reducing its ability to provide services to society [5,6]. The good condition of ecosystems is not considered a service itself; however, it is indispensable as it is an essential condition for human activity.

The ecosystem is degraded when the viability of natural processes and relationships within it are removed or disturbed by anthropogenic activity or the action of natural factors [7]. It is also a process with multiple effects on climate change, biodiversity changes and ecosystem services [8].

Europe is facing a continuing loss of biodiversity, and natural ecosystems are diminishing, especially wetlands, by about 50% [9]. As a member of the European Union (EU), Romania promotes and supports the protection of ecosystems, being part of the United Nations Convention on Biological Diversity (“Convention on Biological Diversity”, 1992). However, like most European countries, Romania is experiencing an increase in its share of degraded ecosystems [10,11].

The restoration of the geological environment and the affected terrestrial ecosystems involves bringing them as close as possible to their natural state, by applying complementary and compensatory cleaning, remediation and/or reconstruction measures and by eliminating any significant risk according to the category of land use. To do this it is necessary to properly evaluate their degradation state and the drivers of this degradation.

Changes in ecosystems are frequently identified on the basis of satellite imagery [12,13]. Most methods focus on biomass evaluation [14,15,16], leaf area index (LAI) [17] or productivity [18,19,20]. The Global Climate Observing System (GCOS), promotes LAI as an essential climate variable (ECV), being a key parameter used in woody ecosystems [21].

In many studies, the health of the ecosystem is analyzed based on GPP (gross primary production) or NPP (net primary production) indices and a vegetation index, such as the normalized-difference vegetation index (NDVI). The high value of these indices does not necessarily mean a good state of health [22], as it may be due to invasive species. In the same way, primary productivity is related not only to variations in CO_2_ in the atmosphere but also to climate change. Globally, productivity has increased in the last 20 years [23].

Machine learning algorithms such as Random Forest (RF) and Support Vector Machine (SVM) are used to identify and monitor vegetation types within forest [24] and grassland ecosystems [25]. Moreover, the SVM image classification method is useful in identifying invasive plant species [26,27], one of the important criteria in the evaluation of grassland ecosystems [27]. Some authors used satellite imagery (Sentinel 2, Landsat 8) and machine learning processes to locate forest treatments over large spatial extents [28].

Another recent analytical framework for the mapping and assessment of ecosystem condition [29] proposed indicators related to environmental pressures (physical and chemical quality) and ecosystem attributes (biological quality) based on a combination of individual metrics.

In October 2010, Japan and the EU member states at the Nagoya Biodiversity Summit signed the Convention on Biological Diversity. In order to achieve its EU biodiversity policy goals and to align with international commitments in the Convention on Biological Diversity, in May 2011, the EU adopted the Biodiversity Strategy to 2020. To achieve Target 2: “Maintain and restore ecosystems and their services”, i.e., the restoration of at least 15% of the degraded ecosystems by 2020, the document proposes that by 2014 each member state should develop a strategic framework for establishing the priorities for the restoration of ecosystems at a national level. In order to respond to the development needs and to contribute to the EU 2020 Strategy, in 2014, the Large Infrastructure Operational Program (LIOP) strategy was elaborated in Romania. Within LIOP, priority axis 4 was established for the promotion of ecological reconstruction projects [30]. Therefore, an assessment and mapping of Romanian degraded ecosystems was necessary. Given the need for composite indicators on ecosystem condition that can reflect the overall quality of an ecosystem asset in terms of its characteristics, the mapping and evaluating of degraded ecosystems in Romania were done.

The results of this paper are based on research started in April 2016, comprising detailed assessment for each type of ecosystem. The databases used were provided by various Romanian and European institutions. These were mainly spatial data, statistical data and satellite images. In the first phase, the degradation criteria and indicators for each type of ecosystem, the degradation classes and the sustainability thresholds were established, as well as the limits and the methodology for ecosystem mapping. In the second phase, the mapping and evaluation of the natural ecosystems were performed, as well as the validation of the results. The integration of all data was achieved and completed in May 2021.

EU Biodiversity Strategy to 2020 set objectives toward mapping and assessing the state of ecosystems from each member state. The target of this strategy was to restore 15% of degraded ecosystems [30] and the current EU-wide Biodiversity Strategy to 2030 aims to protect at least 30% of land and 30% of sea in Europe [31].

The main objectives of our study were as follows: to define and identify the types of natural and semi-natural ecosystems existing in Romania; to develop a nationally applicable methodology for the evaluation of each type of natural and semi-natural ecosystem; and, in this paper, to assess the complete ecosystem’s condition across Romanian territory in order to outline directions for their conservation status.

## 2. Materials and Methods

### 2.1. Study Area

**Figure 1:**
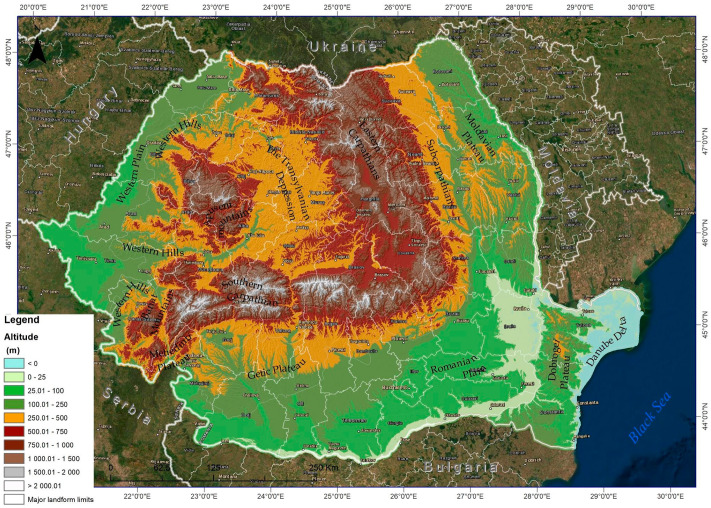
Location of Romania and major landforms.

Romania covers 238,391 km^2^ and is located in southeastern Europe, bordering on the Black Sea and the Danube. The major landforms are concentrically distributed [32], the Transylvanian depression in the center, surrounded by the Carpathian Mountains and hills. Two large plains surround the higher area, namely the Romanian Plain and the Western Plain, to which is added in SE the Danube Delta and Dobrogea Plateau (Figure 1). According to the Köppen–Geiger climate classification, Romania has a temperate continental climate [32]. From a bio-geographical point of view, in Romania, there are five biogeographical regions: Pannonian, Alpine, Continental, Steppic and Black Sea [33].

In a previous study, deliverable of the project Nature in public decisions—N4D, the following types of ecosystems were identified within the Romanian territory: terrestrial (woodland and forest, grassland, cave), freshwater (rivers and lakes), and marine (coastal). Forest ecosystems occupy a total area of about 71,890.84 km^2^, and about 32,357.14 km^2^ of Romanian area is grassland. There are also 339 caves. Rivers are 84,068.17 km in length, lakes represent 2248.28 km^2^, and 1574 km^2^ are coastal ecosystems [10,34].

### 2.2. Data Used

Due to the complexity of each ecosystem, a large number of data were used from different national and international sources available for the entire territory of Romania. Satellite images and satellite imagery products such as MODIS, Landsat and Sentinel 2 were used. Furthermore, vector data such as land use limits (CORINE Land Cover or Land parcel identification system), limits of territorial administrative units (TAUs), limits of protected natural areas, the hydrographic network, the road network, pollution sources, soil types, etc. and statistical data such as livestock and the number of inhabitants were used. The data sets used for each ecosystem are described in Table 1.

### 2.3. Methods

**Figure 2:**
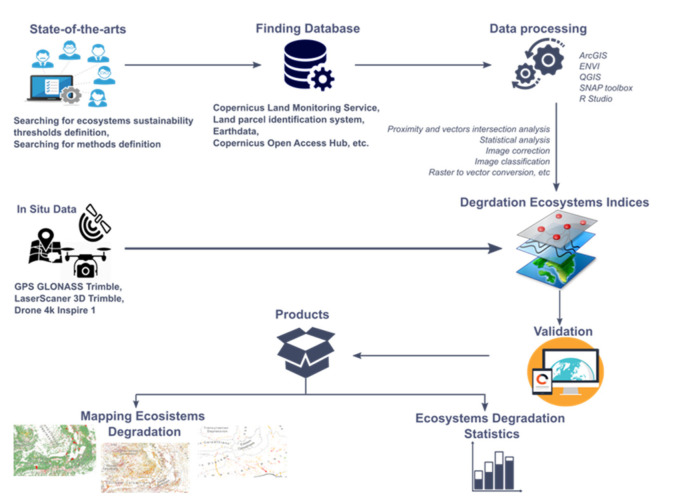
Methodological flow in order to assess the state of ecosystem degradation.

The workflow for the evaluation of the ecosystem’s condition (Figure 2) consisted in three phases. The first phase included state-of-the-art methods for identifying the potential pressures on each ecosystem; searching for ecosystem sustainability threshold definitions; identification of data sets available for the calculation of degradation indicators; data processing and analysis; validation of the applied method. For each ecosystem, a specific assessment methodology was involved. The second phase included correction and adjustment of each indicator used according to the field results; final data processing, field verification and validation. In the final stage, a topological attribute verification was performed.

The methodologies for establishing the level of degradation of the analyzed ecosystems included elements that have an impact on their health and sustainability. The determination of the level of degradation and the classes of degradation also took into account the capacity of ecosystems to support and provide ecosystem services in accordance with their basic functions (Supplementary Material).

The method describing the forest ecosystem’s status was based on the identification of deforested areas using the change detection algorithm between the land use category according to LPIS data [38,54] and the land use in the reference year of 2000, according to the tree canopy cover from Landsat [37]. The conservation status of forest ecosystems was established based on the VCF MODIS [37] product from 2000 to 2013 and the calculation of the linear trend for each pixel [10]. Forest degradation was analyzed by permanent changes in terms of land cover and land use. These changes reduce ecological integrity and health (SER, 2004) affecting the biodiversity and productivity of forests.

The evaluation of the grassland ecosystems was made based on six criteria, each criterion having a specific weight in the final result, Equation (1) [10]. The six criteria refer to the anthropo-zoogenic impact (proximity to localities, proximity to sheepfolds, the total livestock density) [55], stationary conditions (slope) and structural characteristics (invasive species and bare soil/erosions). Each criterion was divided into three classes of values, and each class received a score corresponding to the ecosystem condition, as follows: 0—natural, 1—semi-degraded, 2—degraded [10]. The identification of invasive species and soil or bare soil erosion was based on machine learning algorithms such as Random Forest (RF) and Support Vector Machine (SVM). The degradation state was assigned according to the value of the degradation index (DI). Thus, DI values between 0 and 30 indicated natural grassland ecosystems, between 35 and 60 they represented semi-degraded and between 65 and 180 degraded grassland ecosystems.

DI = (5 × C1) + (20 × C2) + (5 × C3) + (10 × C4) + (50 × C5) + (100 × C6)(1)

where

C1 = proximity to localities (>4 km = natural, 2–4 km = semi-degraded, <2 km = degraded);

C2 = proximity to sheepfolds (>2 km = natural, 0.5–2 km = semi-degraded, <0.5 km = degraded);

C3 = slope (<15° = natural, 15°–30° = semi-degraded, >30° = degraded);

C4 = total livestock density (<±10% = natural, ±10–50% = semi-degraded, >±30% = degraded);

C5 = invasive species (<5% = natural, 5–20% = semi-degraded, >20% = degraded);

C6 = bare soil/erosions (<5% = natural, 5–20% = semi-degraded, >20% = degraded).

Cave ecosystems have been assessed on the basis of the Cave Conservation Index (CCI), which determines the impact of the cave environment and the threats and the vulnerability of the intrinsic characteristics of the caves [56]. CCI is calculated using the score obtained by completing the forms for establishing the impact on the environment of a cave, Rapid Assessment Protocol (RAP-cei) and the score obtained by completing the form to establish the vulnerability of a cave, in order to prioritize conservation and/or restoration actions [57]. Thus, for values between 0 and 34, the cave ecosystem was classified as natural, between 35 and 84 was semi-degraded, and over 85, it was classified as degraded [10].

River ecosystems were assessed based on 13 criteria, grouped into 4 major classes: A. Indicators of the human pressure on riparian areas (anthropization, vegetation cover, human settlements, sewage treatment plants, major pollution sources, transport network, natural protected areas), B. Indicators of substrate of the land adjacent to the watercourse (slope, soil permeability), C. Indicators associated with rivers (human interventions, ecological status of water bodies) and D. Indicators of the morphological complexity of water courses (sinuosity) [58]. Each indicator used in the multicriteria analysis was assigned a weight in the final analysis. The highest weights used in the multicriteria analysis for human pressure on riparian areas were as follows: the anthropization of the adjacent territory of a watercourse, vegetation cover in riparian areas, the presence of major pollution sources, the length of the transport network, human interventions in the riverbanks and the ecological status of water bodies [58].

The assessment of lake ecosystems was performed by combining the potential pollutant load (PPL) developed by [59], wastewater (W)–recreational (R)–agricultural (A)–size (S)–transportation (T)–industrial (I)–cover (C)–pollutant load (WRASTIC) [59] and lake vulnerability (LV) [60], resulting in a new index: WRASTIC-HI index [61,62]. This methodological stage included the analysis of a total of 3189 lakes and their classification by degradation classes.

The assessment of degradation status of the coastal ecosystem was made based on eight indicators, which can be grouped into biological indicators (related to the absence or presence of invasive species), hydro-morphological indicators (related to the presence of wastewater treatment plants, the presence of demographic aggregation poles, shoreline artificialization, shoreline erosion rate) and physical–chemical indicators, data related to transport infrastructure (road infrastructure, navigation channels) and intensity of maritime traffic.

Each indicator was given a score, and the final result was obtained by summing all the scores [10]. Thus, in the terrestrial coastal area, score values ≤4 mean the coastal ecosystem is natural, between 5 and 12 it is semi-degraded and over 13 it is degraded, and in the marine coastal area, score values ≤5 mean the coastal ecosystem is natural, between 5 and 15 it is semi-degraded and over 13 it is degraded.

In order to identify the location of the most degraded ecosystems, the density of each degraded ecosystem and the hotspot analysis based on the Getis–Ord Gi* [63,64] was computed. The hotspot analysis was performed in ArcGIS using the Mapping Clusters tool [65], based on Equation (2) [66].

(2) $$G_{i}^{*}=\frac{\sum_{j=1}^{n}w_{i,j}x_{j}-\bar{X}\;\sum_{j=1}^{n}w_{i,j}}{S\sqrt{\frac{\left[n\sum_{j-1}^{n}w_{i,j}^{2}-{\left(\sum_{j=1}^{n}w_{i,j}\right)}^{2}\right]}{n-1}}}$$

where *G_j*^* statistics is a z-score, *x_j_* is the attribute value for feature *j*, *w_i,j_* is the spatial weight between feature *i* and *j*, *n* is equal to the total number of features and:

(3) $$\bar{X}=\frac{\sum_{j=1}^{n}x_{j}}{n}$$

and

(4) $$S=\sqrt{\frac{\sum_{j=1}^{n}x_{j\;}}{n}-{\left(\bar{X}\right)}^{2}}$$

The density values of each ecosystem were classified into five density classes, and each class was given a score from 1 to 5. Value 1 represents very low density, 2—low density, 3—medium, 4—high, and 5—very high density. The sum of all the layers led to a new layer with the density of degraded ecosystems in Romania and the hotspot analysis based on the Getis–Ord Gi* was performed. The purpose of obtaining a cumulative map of all degraded ecosystems is to highlight their spatial distribution, especially the areas of maximum concentration of degradation, so that structural and non-structural measures to reduce degradation can be taken into account.

## 3. Results and Discussions

The integration of each Romanian ecosystem type assessment indicated that the coastal ecosystem is the most degraded ecosystem, with 86.55% degraded area (1362.32 km^2^). The grassland ecosystem’s evaluation resulted in classification of 38.59% area of grassland as being degraded (12,486.37 km^2^), while 27.64% of river ecosystems were degraded (23,800.22 km length),

A share of 92.92% of cave ecosystems were semi-degraded, followed by 67.67% for lakes and 52.94% for rivers.

Forest ecosystems occupy the largest area of all ecosystems, and a share of 88.54% of this ecosystem was natural, non-degraded. Thus, for the identification of degraded forests, the VCF MODIS sensor was used, which allowed the mapping of forests with a consistency between 30% and 80%, which showed a consistency reduction of over 10%. According to the analysis, only 8.52% of forest ecosystems were degraded (Table 2).

**Figure 3:**
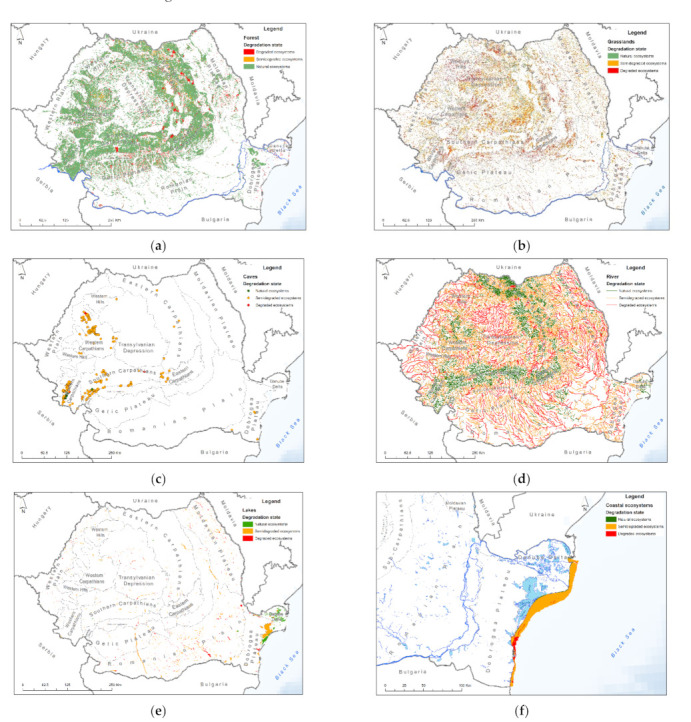
Distribution of ecosystems in Romania: (**a**) forest ecosystems; (**b**) grassland ecosystems; (**c**) cave ecosystems; (**d**) river ecosystems; (**e**) lake ecosystems; (**f**) coastal ecosystems.

The largest area of forest ecosystems was located in the Carpathian Mountains, about 31% in the Eastern Carpathians, 16% in the Western Carpathians and 14% in the Southern Carpathians (Figure 3a). The largest degraded forest ecosystem areas were located in the Eastern Carpathian Mountains, area where deforestation hotspots have been identified in several similar studies [67,68]. The main cause is deforestation resulting in ecosystem loss and fragmentation. Approximately 1124 km^2^ were deforested in the Eastern Carpathians, from which 790 km^2^ were transformed into unproductive land, 652.5 km^2^ into pastures and 177 km^2^ into built-up areas. At the same time, significant forest areas were deforested in the Western Carpathians (approximately 320 km^2^) and the Southern Carpathians (256 km^2^). In the plateau and plain areas, the main cause of degradation was conversion to agricultural land.

The grasslands cover the second largest ecosystem area within Romanian territory (Figure 3b). The largest degraded area of the grassland ecosystem was in the Transylvanian Depression (approximately 2340 km^2^), followed by the Eastern Carpathians with approximately 2220 km^2^ and the Sub-Carpathians with 1433 km^2^. Similar studies on grassland degradation in the Sub-Carpathian area have drawn attention to the degradation rates of grassland in this area and the influence of this process in the manifestation of landslides [69]. These three landforms are concentrated on approximately 50% of the area of degraded grassland ecosystems in Romania. The causes are multiple, from the presence of invasive species such as shrubs to agro-pastoral activities such as excessive grazing.

Most of the cave ecosystems in Romania are located in the Western Carpathians (179 caves), followed by the Southern Carpathians (101 caves) and the Eastern Carpathians (21 caves), (Figure 3c). Most of the caves (50.4%) are located in the Continental biogeographic region, 47.8% in the temperate-continental climatic and 1.8% in the cold semi-arid climate (Dobrogea Plateau).

The assessment of the degree of cave degradation involved the assessment of the environmental and underground impact on the cave ecosystem’s environmental impact and underground impact (slope collapses that led to clogging of entrances or opening of new entrances, water catchments in the karst impluvium, constructions, communication routes in the perimeter of the cave, storage of household waste or other material, excessive and/or disorganized tourism), evaluation of the paleontological deposit—thanatocoenosis (fossil deposit affected by illegal excavations/vandalism, presence of vandalized bioglyphs), archeologic evaluation of the deposit (incisions/drawings with vandalized coal, stone/bone/metal tools destroyed or removed from the archaeological/sedimentological context), assessment of the biodiversity of the underground environment—invertebrate fauna, vertebrate fauna (depending on diversity specific to the fauna of vertebrates and invertebrates in caves).

The analysis showed that approximately 90% of the cave ecosystems were semi-degraded and only 2.66% were degraded.

Most rivers in Romania spring in the Carpathian Mountains, flowing into hilly areas (small rivers) and lowland areas (large rivers). Their condition is influenced by the physical–geographical and socioeconomic characteristics of the areas they pass through. The mountainous areas cover 46.2% of the length of the rivers in Romania, the hill and plateau area constitutes 35.8% and plain and Danube Delta areas 18%.

A preponderance of degraded and semi-degraded river ecosystems were observed in areas with low attitudes, in the plains (Figure 3d). There were approximately 4700 km of degraded rivers in the Romanian Plain and another 5000 km in a semi-degraded state, and 685 km were in a natural state.

In the Transylvanian Depression, approximately 4150 km were classified to a state of degradation, to which were added 5450 km in a state of semi-degradation, leaving only 390 km in a natural state.

Most lake ecosystems are located in the southeastern and eastern part of Romania, respectively, in the Danube Delta (43.1%), the Romanian Plain (22.17%), the Dobrogea Plateau (6.62%) and the Moldavian Plateau (5.39%), (Figure 3e). The largest degraded area of lake ecosystems was located in the Romanian Plain with an area of 177.06 km^2^, representing 35.52% of the lake ecosystems in this area, followed by the Moldavian Plateau with 45.9 km^2^ and the Dobrogea Plateau with 32.16 km^2^. Moreover, in the Dobrogea Plateau and in the Moldavian Plateau were the largest areas of semi-degraded lake ecosystems, 116.48 and 116.09 km^2^, respectively.

The largest degraded areas of coastal ecosystems were in the Periboina–Cap Singol area (71.05 km^2^), the Mangalia Plateau (20.34 km^2^) and the Chituc Grind (18.49 km^2^) (Figure 3f). The largest semi-degraded areas were in the areas Sulina–Periboina (963.83 km^2^), Periboina–Cap Singol (258.11 km^2^) and Eforie–Vama Veche (88.81 km^2^).

**Figure 4:**
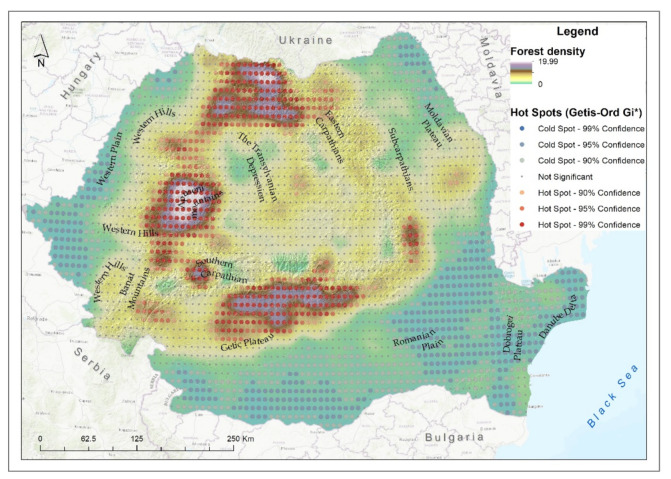
Forest degraded ecosystem density and hotspot analysis.

The highest density of degraded forest ecosystems was identified in the Northeastern Carpathians, the northern group of the Western Carpathians and in the Southern Carpathians and also in the Sub-Carpathians (Figure 4). Moreover, in these areas, the confidence level of the hotspot was over 99%. The extensive forest areas that were deforested in northern Romania, in the Maramureș Mountain area led to landscape degradation and decreased air quality and contributed to the aggravation of the negative effects of torrential floods due to the limited capacity to retain water in the canopy.

**Figure 5:**
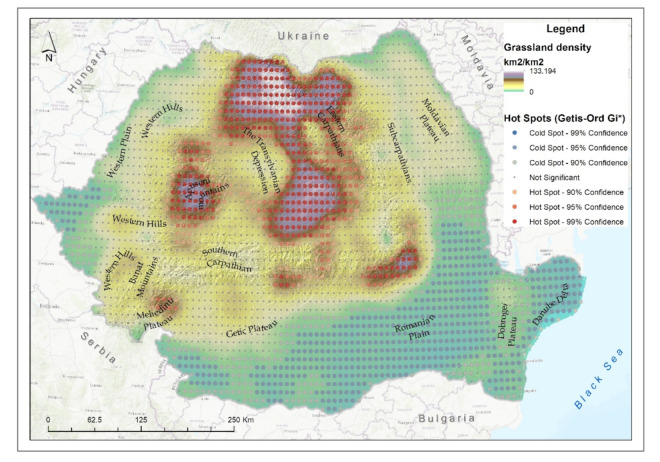
Grassland degraded ecosystem density and hotspot analysis.

The highest density of degraded grassland ecosystems was identified in the Northeastern Carpathians, in the Transylvanian Depression, the north, central-eastern and southern parts, and the confidence level of the hotspot was over 99% (Figure 5). A high density was also observed in the Nordic group of the Western Carpathians.

**Figure 6:**
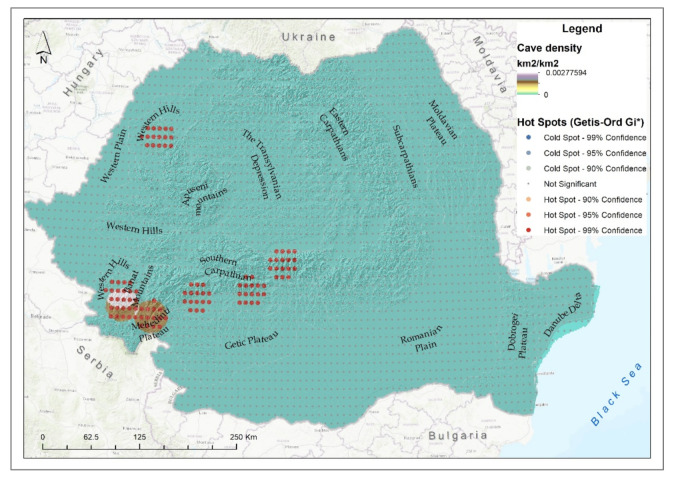
Cave degraded ecosystem density and hotspot analysis.

The density of degraded cave ecosystems in Romania is very low; however, some hotspots can be observed in the south of the Western Carpathians and in the Southern Carpathians (Figure 6). Cold spots identified for mountain areas with a high density of caves in the case of the Apuseni Mountains were due to their low degradation, many of them presenting species from the Red List of Romanian cave fauna [70]. The hotspots identified for the degraded caves were concentrated in the northern Apuseni Mountains, the Banat Mountains, the southern Retezat and Parâng mountains, as well as in Fagaraș (Figure 6). In these areas, there are numerous caves with a high number of tourists, which increases in temperature by up to 2 degrees and increases the pathogenic microorganisms, as determined locally and in studies conducted for Muierilor Cave and Polovragi Cave (from Parâng Mountains) and Urșilor Cave and Meziad Cave (from Apuseni Mountain) [71].

**Figure 7:**
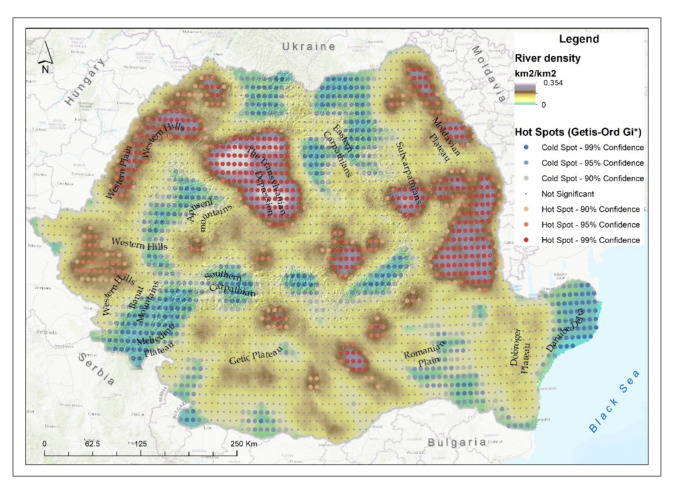
River degraded ecosystem density and hotspot analysis.

The density of degraded river ecosystems was very high in the northern and central part of the Transylvanian Depression, in the northern half of the Western Plain but also in the south of the Moldavian Plateau (Figure 7). Statistically significant hotspots were also registered in the central northern part of the Romanian Plain, in the northeast of the Moldavian Plateau and in the south of the Eastern Carpathians.

The concentration of rivers in the high degradation class in the plain areas (Moldavian Plain located in northeastern Romania, Western Plain and center of the Romanian Plain) is caused by agricultural practices that lead to water pollution due to the use of climatic fertilizers and increase in salinization against the background of increasing average temperatures. In the case of the Transylvanian Depression, a high concentration of degraded areas was also identified. This area is known to be degraded due to the expansion of urban agglomerations but also because of the numerous rural settlements where the sewerage systems do not comply with the environmental regulations in force so that nitrogen pollution is high [72] so that the river degradation class is high.

**Figure 8:**
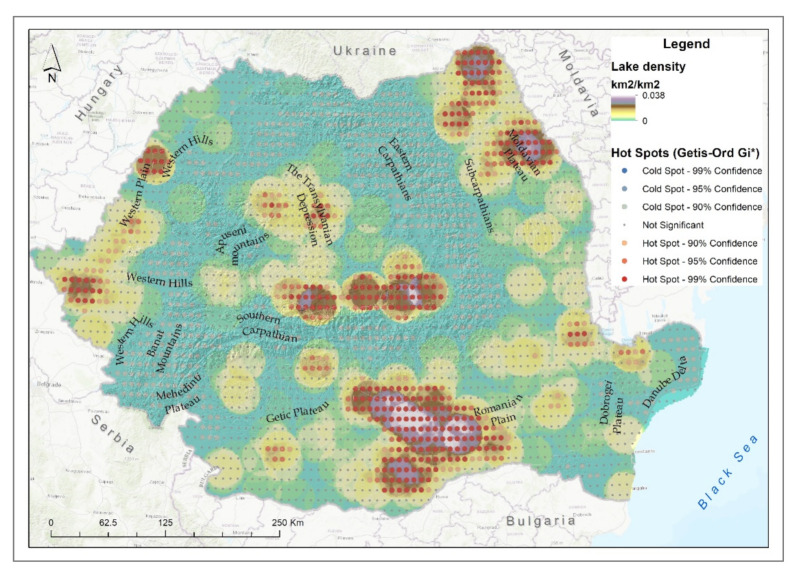
Lake degraded ecosystem density and hotspot analysis.

The highest density of lake ecosystems was identified in the central part of the Romanian Plain, in the south of the Transylvanian Depression and in the north of the Moldavian Plateau (Figure 8). Statistically significant hotspots could also be observed in the Western Plain.

The analysis of the state of degradation of the coastal environment highlighted the areas of expansion of invasive species such as *Ailanthus altissima, Amorpha fruticosa, Elaeagnus angustifolia* and, from the category of marine species, *Rapana venosa* and *Mnemiopsis leidyi* [73].

**Figure 9:**
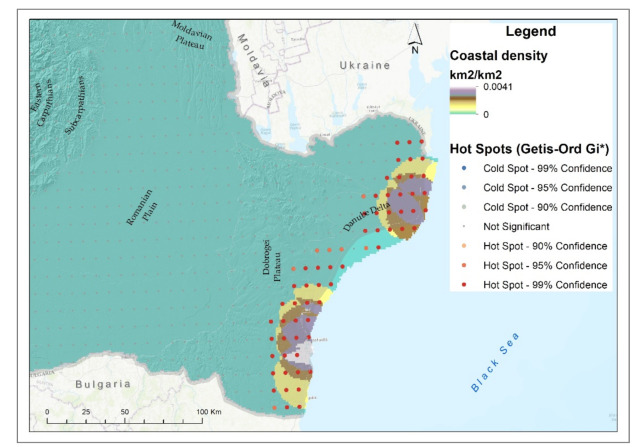
Coastal degraded ecosystem density and hotspot analysis.

The influence of wastewater discharges and the influence of tourist activities was visible for the terrestrial environment of the coastal area, noting in particular the coastal area south of the Dobrogea Plateau, as well as the south of the Danube Delta, territories where the density of tourist resorts is high, thus inducing a negative effect on the studied ecosystem (Figure 9).

**Figure 10:**
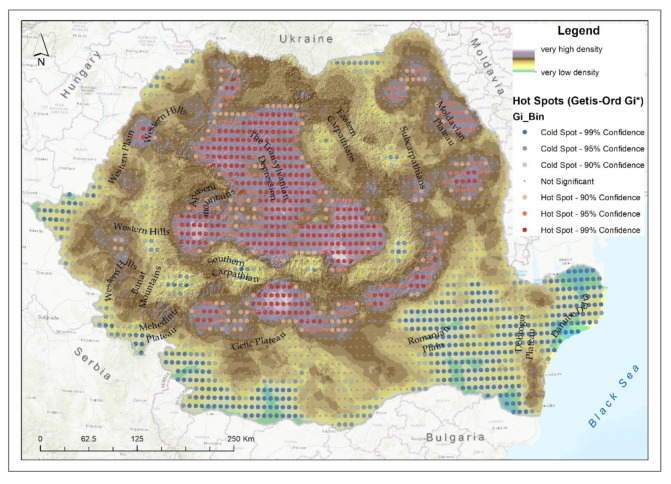
Density of degraded ecosystems and hotspot analysis.

Finally, by combining the densities of all the analyzed ecosystems, the map of the density of degraded ecosystems in Romania was obtained (Figure 10).

This highlights a statistically significant high density in the central part of the country due to the numerous natural meadows that are in a medium and high degradation stage due to the anthropogenic pressure on them, the high number of river segments that are in an advanced stage of degradation due to numerous sources of pollution mainly caused by the lack of septic tanks and the inefficient use of chemical fertilizers in agriculture and the south of the Carpathian Mountains, where, along with the declining forest, there are a high number of degraded meadows but also degraded river segments.

Statistically significant hotspots were also observed in the Moldavian Plateau and in the north of the Western Plain where there was a high number of degraded lakes and rivers as highlighted by the experts involved in the project on-site (Table 3).

Relative operating characteristics (ROC) analysis was used to determine the accuracy of determining the degradation stage for the six types of ecosystems analyzed. The method provides a curve given by a confusion matrix of binary classification according to four possible outcomes: true positive, true negative, false positive and false negative. The results are derived by comparing results of the model with the ground truth survey (GTS), which are established by through field campaigns carried out in spring, summer and autumn for all 6 types of ecosystems with the help of 35 environmental experts from the project, who aimed to identify the state of ecosystems with an emphasis on their degradation. Statistical analyses were conducted using the SPSS software program.

The outcomes are derived by comparing results of the model with the ground truth survey (GTS), approximately 100 points chosen randomly for each type of ecosystem so as to cover all the counties of Romania. The ROC curve is a method that compares true-positive rates against false-positive rates. For each random point, a buffer area of 300 m was analyzed to verify the presence or absence of degradation.

**Figure 11:**
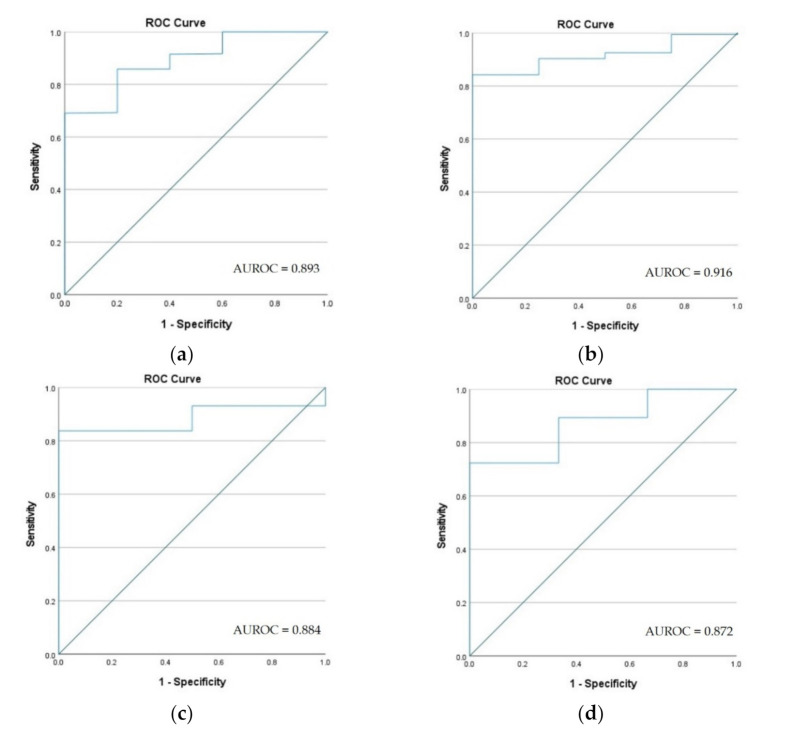

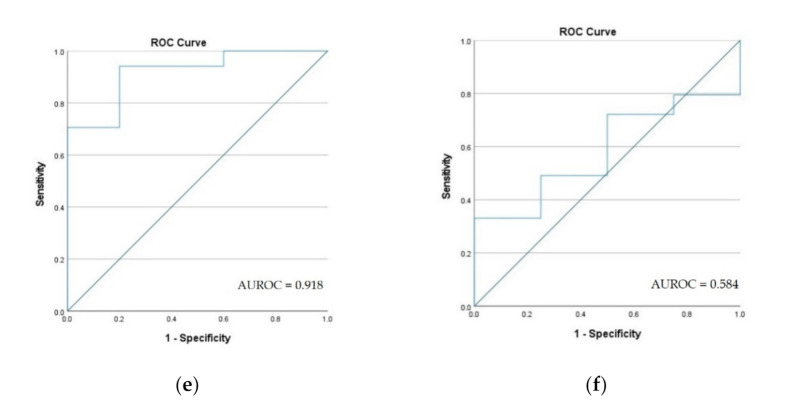
Relative operating characteristics (ROC) curve and AUROC value for degraded ecosystems. (**a**) Forest ecosystems; (**b**) grassland ecosystems; (**c**) cave ecosystems; (**d**) river ecosystems; (**e**) lake ecosystems; (**f**) coastal ecosystems.

Following the analysis of ROC curves for the six types of degraded ecosystems, it can be seen that the models that have a high degree of representativeness for the analyzed problem were those that focused on identifying degraded ecosystems (characterized by a value of the area of under the ROC cure of 0.916) and the model for determining the degraded lakes (characterized by a value of the area under the ROC cure of 0.918) (Figure 11).

In the case of the models that targeted the ecosystems of forests, caves and rivers, lower values were calculated but located above the limit of 0.800, considered a threshold value in order to frame the results in the category of strong and moderate models [74]. However, these values are justified, taking into account the diversity of the causes of degradation as well as their uneven distribution at the national level [75]. The model with a low validation rate was the coastal ecosystems model for which improvements can be made in future studies, aimed at lowering the distinction between the distribution of disturbing factors on land and water and their dispersion with distance from shore [76]. However, we considered that, for the present study, we should keep all six models so that the final map of cumulative degradation of all ecosystems can be made at the national level and draw attention to hotspots that require detailed studies or case studies to analyze in time the current situation of degradation.

## 4. Conclusions

Identifying degraded ecosystems is a key element of the ecological reconstruction strategy. In this sense, their analysis contributes to a better understanding of the mechanisms that have led to changes in the structure and functioning of ecosystems, with a direct impact on ecosystem services. The conceptual approach based on the mapping and assessment of ecosystem services contributes significantly to the development of an integrated vision of ecological reconstruction.

Represented by the variety of ecosystems, species and genes, the biodiversity in Romania is the national natural capital, being an integral part of sustainable development, by providing goods and services such as food, carbon sequestration and redistribution of marine and terrestrial water, which underlie prosperity, economic development, social welfare and quality of life.

Human activities are assessed in terms of direct or indirect impact on the components of biological diversity in order to apply appropriate measures to minimize adverse effects, reconstruction, rehabilitation and remediation of affected ecosystems.

Considering the fact that for all 6 types of ecosystems a group of 35 environmental professionals performed field studies and identified the state of the ecosystems with an emphasis on their degradation, we consider that the database used reaches the degree of detail necessary to draw general conclusions in terms of the concentration of degraded areas in Romania. Following the hotspot analysis, it was identified that the largest degraded surfaces are the coastal ones (49.80%), followed by the grassland ecosystems (38.59%) and the cave ecosystems (2.66%), while the degraded rivers ecosystems are degraded by a proportion of 27.64%, degraded forest ecosystems by 8.52%, and degraded lakes ecosystems by 14.05%. Relative operating characteristics (ROC) analysis highlights that the models that have a high degree of representativeness are the grassland ecosystems (characterized by a value of the area of under the ROC curve of 0.916) and lake ecosystems (with ROC cure value of 0.918). Ecosystems characterized by a great diversity of the causes of degradation as well as their uneven distribution at the national level such as the ecosystems of forests, caves and rivers with a ROC value above the limit of 0.800.

The degradation of a particular ecosystem must be assessed by the characteristics of the ecosystem to be restored. The methodology for assessing the degree of degradation of ecosystems is based on a series of activities, criteria, methods and procedures for estimating the values of the parameters that indicate the state of these ecosystems. Therefore, it is important to discover the natural processes that take place in the system and to analyze the changes produced by the impact of anthropogenic activities. Conservation status assessment and monitoring consists of identifying direct or indirect risks and assessing the degree of habitat threat.

The study carried out on the changes that occurred in the natural environment on the Romanian territory shows how the deterioration and pollution of the areas is directly related both to the industrial activities in the area and to the inevitable climatic changes and other natural phenomena.

In conclusion, the highest density of degraded ecosystems in Romania is located in the central part, in the Transylvanian Depression and south of the Carpathian Mountains, in the Sub-Carpathians. The main factors that led to the degradation of ecosystems in Romania were anthropogenic but also natural.

This comprehensive study is an important step in the field of ecological reconstruction in Romania, as the starting point for future studies and supplementary rehabilitation actions.

## Figures and Tables

**Table 1:** Used geo-database.

Major Ecosystem Category	Ecosystem Type for Mapping and Assessment	Name of Datasets	Resolution/Minimum Mapping Unit	Time Reference	Source
Terrestrial	Forest	Land-parcel identification system (LPIS)	NA	2013	Land-parcel identification system from Romania [35]
		Vegetation Continuous Fields (MOD44B)	250 m	2000–2013	LAADS DAAC [36]
		Hansen Global Forest Change	30 m	2000–2013	Global Forest Change [37]
	Grassland	Land-parcel identification system (LPIS)	NA	2017	Land-parcel identification system from Romania [38]
		Limits of territorial administrative units (TAUs)	NA	2016	National Agency for Cadastre and Land Registration of Romania [39]
		Digital surface model (EU-DEM)	25 m	2011	Copernicus Land Monitoring Service [40]
		Livestock numbers and types from TAUs	NA	2010	National Statistics Institute of Romania [41]
		European Settlement Map	10 m	2010–2013	Copernicus Land Monitoring Service [42]
		Sentinel 2 images	10 m	2015–2017	Copernicus Open-Access Hub [43]
	Cave	Natura 2000 (N2K)	NA	2017	Ministry of Environment, Waters and Forests [44]
		Orthophotos	0.5 m	2016	National Agency for Cadastre and Land Registration of Romania [39]
		CORINE Land Cover (CLC 2012)	100 m	2011–2012	Copernicus Land Monitoring Service [45]
		European Settlement Map	10 m	2010–2013	Copernicus Land Monitoring Service [42]
		European catchments and Rivers network system (ECRINS—dams on rivers)	1:250,000	2012	European Environment Agency [46]
		Roads and railways	NA	2016	Open Street Map [47]
Fresh water	Rivers	EU-Hydro—River Network	1 ha	2006–2012	Copernicus Land Monitoring Service [48]
		European catchments and Rivers network system (ECRINS—dams on rivers)	1:250,000	2012	European Environment Agency [46]
		Digital surface model (EU-DEM)	25 m	2011	Copernicus Land Monitoring Service [49]
		CORINE Land Cover (CLC 2012)	100 m	2011–2012	Copernicus Land Monitoring Service [45]
		European Settlement Map	10 m	2010–2013	Copernicus Land Monitoring Service [42]
		Riparian Zones 2012—Land Use Land Cover	0.5 ha	2011–2013	Copernicus Land Monitoring Service [49]
		Urban Wastewater Treatment (Water-based UWWTD)	NA	2015	European Environment Agency [50]
		Natura 2000 (N2K)	NA	2017	Ministry of Environment, Waters and Forests [44]
		Roads and railways	NA	2016	Open Street Map [47]
		Romania soils map	NA	2017	National Institute of Research and Development for Pedology, Agro-chemistry and Environmental Protection [51]
		Number of inhabitants from TAUs	NA	2017	National Statistics Institute of Romania [52]
		Limits of territorial administrative units (TAUs)	NA	2016	National Agency for Cadastre and Land Registration of Romania [39]
	Lakes	Permanent water bodies	20 m	2012	Copernicus Land Monitoring Service
		CORINE Land Cover (CLC 2012)	100 m	2011–2012	Copernicus Land Monitoring Service [45]
		Digital surface model (EU-DEM)	25 m	2011	Copernicus Land Monitoring Service [40]
		Roads and railways	NA	2016	Open Street Map [47]
		Exploitation areas of natural resources	NA	2017	National Agency for Mineral Resources
		Natura 2000 (N2K)	NA	2017	Ministry of Environment, Waters and Forests [44]
		Romania soils map	NA	2017	National Institute of Research and Development for Pedology, Agro-chemistry and Environmental Protection [51]
		Urban Wastewater Treatment (Waterbased UWWTD)	NA	2015	European Environment Agency [50]
		Limits of territorial administrative units (TAUs)	NA	2016	National Agency for Cadastre and Land Registration of Romania [39]
		Landsat 8	30 m	2016	[53]
Marine	Coastal	CORINE Land Cover (CLC 2012)	100 m	2011–2012	Copernicus Land Monitoring Service [45]
		Digital surface model (EU-DEM)	25 m	2011	Copernicus Land Monitoring Service [40]
		Roads and railways	NA	2016	Open Street Map [47]
		Urban Wastewater Treatment (Waterbased UWWTD)	NA	2015	European Environment Agency [50]
		European Settlement Map	10 m	2010–2013	Copernicus Land Monitoring Service [42]
		Landsat 8	30 m	2016	[53]
		Orthophotos	0.5 m	2016	National Agency for Cadastre and Land Registration of Romania [39]

**Table 2:** Summary of ecosystem assessment results.

Ecosystem	Natural	%	Semi-Degraded	%	Degraded	%	Total
Forest (km^2^)	63,651.34	88.54	2115.27	2.94	6124.23	8.52	71,890.84
Grassland (km^2^)	7080.05	21.88	12,790.72	39.53	12,486.37	38.59	32,357.14
Cave (no.)	15	4.42	315	92.92	9	2.66	339
Lake (km^2^)	410.95	18.28	1521.41	67.67	315.91	14.05	2248.28
River (km)	16,320.52	19.41	44,508.82	52.94	2238.82	27.64	84,068.17
Coastal (km^2^)	42.5	2.7	1362.32	86.55	169.17	49.8	1574

**Table 3:** Classification of relief units in Romania by degradation classes.

Degradation.	Low (1–11)		Medium (11–15)		High (15–22)	
Relief Units	kmp	%	kmp	%	kmp	%
Eastern Carpathians	2778.9	8.1	23,597.6	68.7	7949.8	23.2
Southern Carpathians	2759.6	19.5	7120.7	50.3	4265.8	30.2
Banat Mountains	1747.8	25.1	4448.4	63.8	780.3	11.2
Sub-Carpathians	215.5	1.3	6884.7	41.5	9473.0	57.2
Apuseni Mountains	275.6	2.6	5976.2	56.1	4399.7	41.3
Transylvanian Depression	0.0	0.0	2079.0	8.2	23,183.6	91.8
The Western Hills	951.6	7.4	8034.8	62.7	3825.7	29.9
Mehedinti Plateau	18.1	2.3	519.5	65.1	259.7	32.6
The Getic Plateau	1879.4	13.6	7247.7	52.6	4654.9	33.8
The Plateau of Moldova	1215.9	5.3	12,506.0	54.8	9091.9	39.9
Western Camp	4059.4	25.3	10,012.9	62.3	2001.9	12.5
The Romanian Plain	24,664.9	50.6	22,218.3	45.5	1903.2	3.9
Dobrogea Plateau	6587.6	65.1	3424.2	33.8	105.5	1.0
The Danube Delta	4403.1	99.0	43.1	1.0	0.0	0.0

Low, medium and high classes were obtained using a natural-break classification of the density of degraded raster ecosystems presented in Figure 10.

## Supplementary Materials

The following are available online at https://www.mdpi.com/article/10.3390/ijerph182111416/s1, Supplementary: Ecosystem services and type and sources of degradation used in this study.
