# Supplementary material for: Applying a Complex Integrated Method for Mapping and Assessment of the Degraded Ecosystem Hotspots from Romania
Source: Int J Environ Res Public Health. 2021 Oct 29;18(21):11416. doi: 10.3390/ijerph182111416 (PMC8583292; doi:10.3390/ijerph182111416)
Supplement: Supplementary file 1 [file ijerph-18-11416-s001.zip › ijerph-1405638-supplementary.pdf]

International Journal of
Environmental Research and Public Health

MDPI

Supplementary. Ecosystem services and type and sources of degradation used in this study.

Ecosystem services [76]	Ecosystems analyzed in the study	Types and sources of degradation taken into account in the working methodology [10]
Support:<br>- for the circulation of nutrients<br>- for pedogenesis<br>- for the basic production of the elements<br><br>For Supply:<br>- with nutrients<br>- with water<br>- with wood<br>- with fuel<br><br>Regularization / control<br>- of the climate<br>- of floods<br>- of diseases<br>- water filtration<br><br>Cultural aspects<br>- aesthetics<br>- spiritual<br>- educational<br>- recreation	1. Rivers	* degree of anthropogenicity adjacent to the body of water (dense urban areas, exploitation, industrial areas, irrigated or non-irrigated agricultural land, presence of orchards, vineyards)<br>* vegetation characteristics in the riparian zone (the presence of vegetation of what kind and in what percentage)<br>* Demographic density of settlements along the river / segments<br>* the presence of major sources of pollution (industrial spaces, livestock complexes, landfills, etc.)<br>* proximity to transport infrastructure<br>* the presence of protected natural areas on the river course / segments<br>* anthropogenic interventions in the riverbed (dams, dams)<br>* flooding capacity<br>* the state of the ecological balance of the water bodies, etc.
	2. Coastal area	* invasive species, the presence of wastewater, the impact of tourist activities, continental and maritime transport infrastructure, shoreline artificialization works, geomorphological phenomena, equipotential areas, etc.
	3. Forest	* transformation of the land into another category of use and the degree of canopy coverage as an indicator of declining tree density
	4. Lakes	* the presence of wastewater through aggregation cores, treatment plants<br>* recreational activities (water sports, access, tourist infrastructure)<br>* agricultural activities (permanent irrigation, agricultural area used within the receiving basin)<br>* extension of the surface of the supply basin<br>* proximity of transport routes (railways, roads)<br>* industrial activities (industrial units, operating perimeters)<br>* percentage of vegetation cover<br>* geomorphological aspects with potential impact on the quality of lake ecosystems (slope, exposure, soil permeability, etc.)
	5. Meadows	* proximity to localities<br>* proximity to sheepfolds<br>* slope<br>* the average potential load with animals

		* degree of coverage with invasive woody / grassy vegetation<br>* degree of soil cover / erosion
	6. Caves	* the presence of biodiversity elements<br>* litho-pedological characteristics<br>* the characteristic of the vegetation at the mouths of the caves<br>* the presence of the anthropic phenomenon
